# The Role of Autophagy in Skeletal Muscle Diseases

**DOI:** 10.3389/fphys.2021.638983

**Published:** 2021-03-25

**Authors:** Qianghua Xia, Xubo Huang, Jieru Huang, Yongfeng Zheng, Michael E. March, Jin Li, Yongjie Wei

**Affiliations:** ^1^Affiliated Cancer Hospital and Institute of Guangzhou Medical University, Guangzhou, China; ^2^Center for Applied Genomics, The Children’s Hospital of Philadelphia, Philadelphia, PA, United States

**Keywords:** autophagy, AMPK, mTOR, muscle cell homeostasis, transcriptional regulation, skeletal muscle diseases

## Abstract

Skeletal muscle is the most abundant type of tissue in human body, being involved in diverse activities and maintaining a finely tuned metabolic balance. Autophagy, characterized by the autophagosome–lysosome system with the involvement of evolutionarily conserved autophagy-related genes, is an important catabolic process and plays an essential role in energy generation and consumption, as well as substance turnover processes in skeletal muscles. Autophagy in skeletal muscles is finely tuned under the tight regulation of diverse signaling pathways, and the autophagy pathway has cross-talk with other pathways to form feedback loops under physiological conditions and metabolic stress. Altered autophagy activity characterized by either increased formation of autophagosomes or inhibition of lysosome-autophagosome fusion can lead to pathological cascades, and mutations in autophagy genes and deregulation of autophagy pathways have been identified as one of the major causes for a variety of skeleton muscle disorders. The advancement of multi-omics techniques enables further understanding of the molecular and biochemical mechanisms underlying the role of autophagy in skeletal muscle disorders, which may yield novel therapeutic targets for these disorders.

## Introduction

Skeletal muscles, a type of highly organized and specialized tissue in vertebrates, make up about 40% of total body mass and play a central role in diverse activities, such as locomotion, macromolecule turnover and storage, energy metabolism, and oxygen consumption (Tortora and Anagnostakos, 1987; Frontera and Ochala, 2015). Under starvation conditions, skeletal muscles serve as a significant internal source of nutrients, energy, and cellular building blocks. Additionally, the different types of activities that skeletal muscles are involved in, especially prolonged or high-intensity exercises, generate reactive oxygen species (ROS), which results in the damage of macromolecules such as nucleic acids, proteins, lipids, and cellular components (Neel et al., 2013). Molecular signaling pathways and cellular processes in skeletal muscles are shaped to efficiently cope with such cellular damage as well as other types of injury.

The autophagy signaling pathway is essential for energy generation/consumption and macromolecule turnover processes in skeletal muscles. Abnormal autophagy in muscles results in cellular alterations such as mitochondrial damage, endoplasmic reticulum stress, impaired sarcomeric-protein turnover, and cell death (Bonaldo and Sandri, 2013), leading to the development of various types of skeletal muscle disease. The critical roles of autophagy in skeletal muscles have been gaining more attention over the past two decades. In this review, we discuss the physiological function of autophagy in maintaining cellular homeostasis of skeletal muscle and the role of autophagy in muscle disorders.

## The Evolutionarily Conserved Autophagy Pathway

The term “autophagy,” which means “self-eating,” is a highly conserved process from yeast to plants and animals (Ohsumi, 2014; Levine and Kroemer, 2019). Genetic studies in yeast have identified essential Autophagy-related genes (ATG) (Tsukada and Ohsumi, 1993; Thumm et al., 1994; Harding et al., 1995; Titorenko et al., 1995; Yuan et al., 1997; Sakai et al., 1998; Mukaiyama et al., 2002; Parzych et al., 2018). A total of 42 *ATG* genes have been identified (Klionsky et al., 2016; Parzych et al., 2018; Delorme-Axford and Klionsky, 2019); they are highly conserved from yeast to human. Studies using reverse genetic tools have further illustrated the physiological and pathophysiological roles of autophagy in multiple cellular events in higher organisms, including human cells. The autophagy pathway comprises a series of highly organized sequential steps responsible for recruiting and degrading misfolded proteins and the recycling of the decomposition products.

**FIGURE 1:**
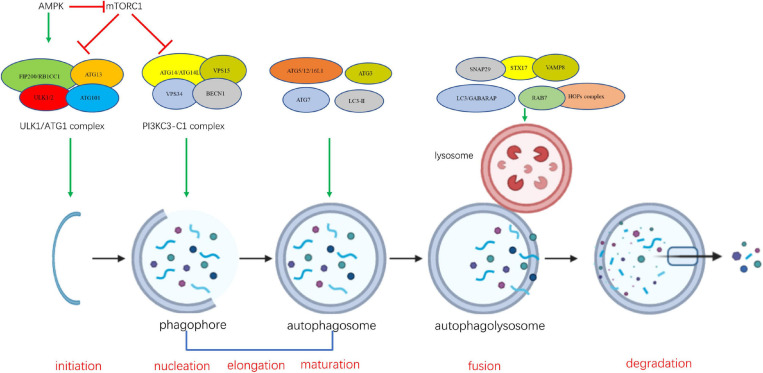
The sequential steps of autophagy involve the participation and interaction of the ATG proteins.

A series of dynamic membrane events contribute to autophagosome formation, involving several steps of morphological change in the cell (Parzych and Klionsky, 2014). The sequential steps of autophagy involve the participation and interaction of the ATG proteins Figure 1. The ULK/Atg1 complex is responsible for the initiation of autophagosome formation. This complex consists of five members in yeast (Atg1, Atg13, Atg17, Atg29, and Atg31) and four members in mammals (ULK1/2, ATG13, FIP200/RB1CC1, and ATG101) (Papinski and Kraft, 2016). ULK1/2 (Atg1 in yeast) is the only core protein with serine/threonine kinase activity in the autophagy signaling pathway. Autophagy signaling is mediated by activation of the ULK/Atg1 complex prior to autophagosome assembly. The ULK/Atg1 complex acts as a bridge between the upstream nutrient or energy integrator mTOR and the downstream ATG proteins involved in autophagosome formation, phosphorylating a variety of downstream proteins. It is believed that downstream ATG proteins are not necessary for membrane recruitment of ULK/Atg1 complex at the initiation stage (Suzuki et al., 2007; Koyama-Honda et al., 2013).

The formation of the class III phosphatidylinositol-3 kinase complexes I (PI3KC3-C1) is an essential event at the nucleation stage, which follows the formation of ULK/Atg1 complex (Kihara et al., 2001; Itakura et al., 2008). Vesicle nucleation leads to formation of the isolation phagophore (Levine and Kroemer, 2008). PI3KC3-C1 is composed of four components including the catalytic subunit PIK3C3 (also known as VPS34), BECLIN 1 (mammalian homolog of yeast Atg6), PIK3R4 (phosphoinositide-3-kinase regulatory subunit 4, also known as VSP15), and Atg14/ATG14L (Autophagy-Related Protein 14-Like Protein) (Kihara et al., 2001; Itakura et al., 2008; Matsunaga et al., 2009; Levine and Kroemer, 2019). During the initiation of autophagy, PI3KC3-C1 is activated and recruited to sites of phagophore nucleation on the endoplasmic reticulum (ER) and mitochondria to convert phosphatidylinositol (PI) into phosphatidylinositol 3-phosphate (PI3P) (Funderburk et al., 2010; Fan et al., 2011; Hamasaki et al., 2013). Although a number of studies have demonstrated that Atg14-containing complex PI3KC3-C1 is involved in the formation of autophagosome, several reports suggested that UVRAG-PI3KC3-C2 complex is critical for Vps34 function on endolysosomal and autophagolysosomal maturation (Itakura and Mizushima, 2009; Matsunaga et al., 2009; Nezis et al., 2014; Levine and Kroemer, 2019). PI3KC3-C2 shares PI3KC3, BECLIN1, and PIK3R4 with PI3KC3-C1, but contains a UVRAG (UV radiation resistance associated gene protein) instead of ATG14L (Itakura et al., 2008).

Vesicle nucleation is followed by the elongation and expansion of phagophore in the cytoplasm. The phagophore becomes a cup-shaped double membrane structure and begins to surround cytoplasmic material (Lamb et al., 2013). Two ubiquitin-like proteins, autophagy-related 12 (ATG12) and microtubule-associated protein 1 light chain 3 alpha/beta (MAP1LC3A/MAP1LC3B, LC3), a human homolog of yeast Atg8 (Ohsumi and Mizushima, 2004; Nakatogawa, 2013, 2014), play essential roles in the elongation and expansion of phagocytic membranes. ATG12 is conjugated to autophagy-related 5 (ATG5), mediated by E1 ubiquitin ligase-like conjugating enzyme autophagy-related 7 (ATG7) and E2 ubiquitin ligase like conjugating enzyme autophagy-related 10 (ATG10), and then the interacts with autophagy-related 16 (ATG16) non-covalently (Romanov et al., 2012; Walczak and Martens, 2013). LC3 is cleaved by the autophagy-related 4 (ATG4) cysteine peptidase at the C-terminal end to produce cytoplasmic LC3-I, which is also linked to phosphatidylthanolamine (PE) in a ubiquitin-like reaction that requires ATG7 and autophagy-related 3 (ATG3) to form LC3-phosphatidylethanolamine conjugate (LC3-II). LC3-II, in this lipid form, is integrated into the autophagosomal membrane and has been regarded as an autophagosomal marker (Fujita et al., 2008; Burman and Ktistakis, 2010).

Phagophore closure during the maturation stage results in sequestration of cytoplasmic component and formation of the autophagosome. As maturation proceeds, the autophagosome fuses with endosomes and vacuoles (in yeast and plant) or lysosomes (in metazoan cells), forming the autophagolysosome and leading to the degradation of the inner membrane and its contents. When the degradation is complete, the autophagolysosome becomes a residual body. The resulting breakdown products such as amino acids and fatty acids are sent back to the cytoplasm and are reused for cellular metabolism, providing an internal source of energy generation and building blocks for catabolism (Ohsumi, 2014; Sakakibara et al., 2015). The underlying mechanism by which autophagosome fusion is regulated is not clear. Recent studies suggest that Atg8 proteins may act as a master controller for the final fusion stages of autophagy (Nguyen et al., 2016; Yu and Melia, 2017; Kriegenburg et al., 2018). Several other proteins, such as SNAREs (syntaxin 17, SNAP29 and VAMP8), and tethering factors (HOPS complex, and the Rab GTPase, RAB7) are required for the fusion of the autophagosomal membrane with the lysosome (Itakura et al., 2012; Balderhaar and Ungermann, 2013; Schaaf et al., 2016; D’Agostino et al., 2017).

## Molecular Regulation of the Autophagy Pathway in Skeletal Muscles

**FIGURE 2:**
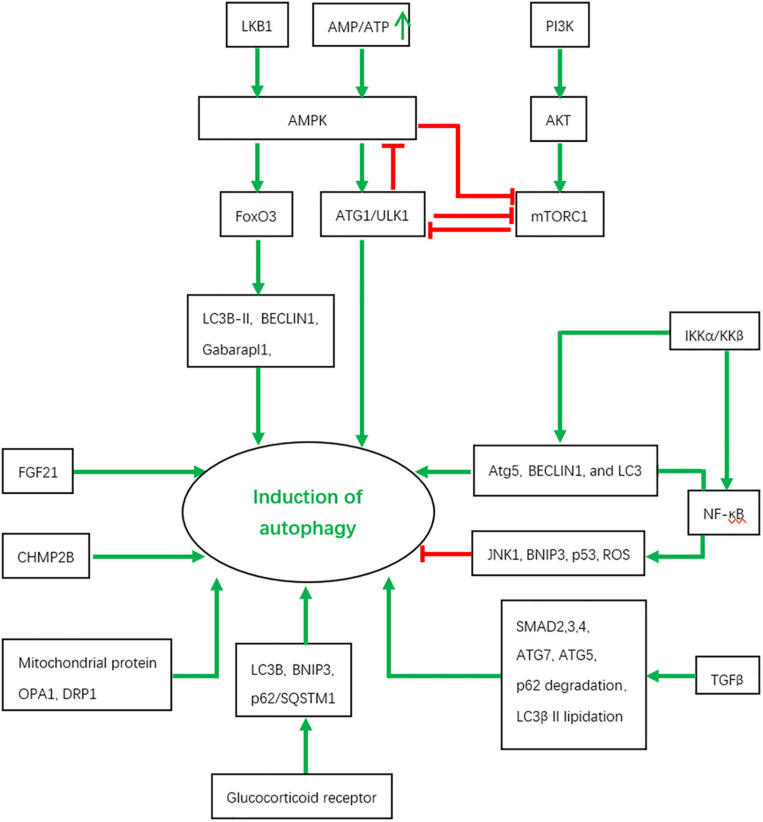
The regulation of autophagy by various signaling pathways and the interactions between them. Red color represents inhibition and green color indicates activation.

In skeletal muscles, autophagy is under the tight regulation of several signaling inputs and interacts with other signaling pathways Figure 2.

### AMPK and mTORC1

The AMPK and mammalian target of rapamycin complex 1 (mTORC1) signaling pathways, as central players in cell survival, proliferation and metabolism, have numerous interconnections with the core genes of the autophagy pathway.

AMPK acts as an energy sensor to monitor changes in the intracellular level of ATP, which is particularly critical in muscles where high rates of energy consumption occur. The growth of skeletal muscles depends on the balance between protein catabolism and anabolism. Atrophy occurs when the rate of muscle protein breakdown is higher than that of muscle protein synthesis. Activated by a rising AMP/ATP ratio, AMPK up-regulates ATP-producing catabolic pathways while suppresses ATP-consuming biosynthetic processes (Winder et al., 2000; Viollet et al., 2003; Wang et al., 2011). AMPK activity is also under the regulation of Liver kinase B1 (LKB1) and mTORC1 through a series of phosphorylation events in skeletal muscles, as revealed in cell culture and model organism studies (Bolster et al., 2002; Sakamoto et al., 2005; Koh et al., 2006; Williamson et al., 2006; Thomson et al., 2007, 2010; Ou et al., 2018).

AMPK modulates autophagy through at least two mechanisms in skeletal muscle. AMPK activation in mouse skeletal muscle results in a relocalization of transcription factor Forkhead box O3a (FoxO3a) to the nucleus where it induces the expression of autophagy-related genes including LC3B-II, Gabarapl1, and BECLIN1 through transcriptional regulation which we will discuss in more detail below, leading to autophagy (Sanchez et al., 2012). In addition, under nutrient starvation conditions, mammalian AMPK directly phosphorylates ULK1 at Ser 317 and Ser 777, promoting the initiation of autophagy which subsequently provides energy and nutrition (Kim et al., 2011; Ljubicic and Jasmin, 2013). AMPK activation releases ULK1 from the complex composed of AMPK, mTORC1, ULK1, FIP200, and Atg13, leading to autophagy activation (Hosokawa et al., 2009). These results suggest that a basal level of autophagy is required to degrade misfolding proteins and damaged organelles to maintain homeostasis under normal nutritional conditions, while autophagy is upregulated by AMPK activation to degrade proteins as a source of alternative nutrients and energy under stress responses such as starvation and exercise (Sanchez et al., 2012).

The mTOR signaling pathway is an evolutionarily conserved pathway which controls multiple cellular processes, including metabolism, protein synthesis, ribosome biogenesis, cell growth, differentiation, and autophagy (Wullschleger et al., 2006; Laplante and Sabatini, 2012). mTOR is classed into two distinct complexes, named mTORC1 and mTORC2 (Laplante and Sabatini, 2012). mTORC1 consists of raptor (regulatory associated protein of mTOR), mLST8 (the mammalian lethal with SEC13 protein 8), PRAS40 (the 40 kDa proline-rich Akt substrate), and DEPTOR (the DEP domain-containing mTOR-interacting protein), and is sensitive to rapamycin (Wullschleger et al., 2006). mTORC2 shares DEPTOR and mLST8 with mTORC1 and includes the distinct components rictor (rapamycin insensitive companion of mTOR) and mSIN1 (mammalian stress-activated map kinase-interacting protein 1) (Jacinto et al., 2004; Sarbassov et al., 2004).

Several studied have demonstrated that the mTORC1 signaling is essential for muscle function. Mice lacking muscle specific tuberous sclerosis complex (TSC) showed sustained activation of mTORC1 and unchanged levels of LC3I and LC3II under fed, basal, and starved conditions, suggesting constitutive and starvation-dependent autophagy is blocked. This impaired autophagy gives rise to a severe, late-onset myopathy. Rapamycin treatment restores autophagy and alleviates the myopathic phenotype of the mice. Although FoxO3 is activated, constitutive and starvation-induced autophagy is blocked by mTORC1-mediated inhibition of ULK1. mTORC1 phosphorylates ULK1 at several sites, such as Ser 757, which prevents interaction between ULK1 and AMPK (Kim et al., 2011; Bento et al., 2016). Paradoxically, abolishment of mTORC1 activity by deletion of raptor also induces autophagy, even though FoxO-dependent transcription of autophagy genes is reduced. These data demonstrate that mTORC1 is another upstream regulator for autophagy induction in skeletal muscle (Castets et al., 2013).

Other studies also show the feedback of ULK1 on AMPK and mTORC1. ULK1 can directly phosphorylate raptor and negatively regulate either mTORC1 activity or substrate binding (Alers et al., 2012). All three subunits of AMPK (AMPKα1, -β2, and -γ1) can serve as direct substrates of ULK1 and ULK2. Through such phosphorylation events, ULK1 confers negative regulation of AMPK kinase activity (Alers et al., 2012). Thus, in addition to being a regulatory target of AMPK and mTORC1, the autophagy pathway constitutes a negative regulatory feedback loop to both signaling pathways and maintains a finely balanced state of cellular homeostasis.

### Transcriptional Regulation

As previously mentioned, transcriptional regulation is a key component of autophagy regulation in skeletal muscle. A large number of studies have demonstrated that autophagy is under the control of multiple transcription factors such as Forkhead box O3 (FoxO3), nuclear factor κB (NF-κB), glucocorticoid receptors (GR), and SMAD. Autophagy genes are targets of these transcription factors under metabolic stress conditions.

#### FoxO3

FoxO3 functions as an activator of the transcription of autophagy genes *ATG4, ATG8B, ATG12, LC3, BECLIN1, BNIP3, VPS34, ULK1*, and *ULK2* in skeletal muscle (Mammucari et al., 2007; Zhao et al., 2007; Sanchez et al., 2012; Di Malta et al., 2019). Importantly, FoxO3 plays a necessary and sufficient role in muscle atrophy through its effect on autophagy. Inhibition of Bnip3 largely blocks autophagy induced by FoxO3 (Sandri et al., 2004; Mammucari et al., 2007). A study using muscle-specific FoxO1,3,4–/– mice has identified that 29 of the 63 atrophy-related genes are controlled by FoxO under the fasting condition. The induction of autophagy-related genes like *LC3, Gabarapl, Bnip3* etc. were abolished in the FoxO1,3,4–/– mice. Consistently, autophagy is severely impaired in these mice.

It is well known that the ubiquitin-mediated proteasome system (UPS) and autophagy/lysosome system are two major mechanisms for degradation of damaged or misfolded proteins. It has been found that both the autophagy-lysosome and the ubiquitin–proteasome system are under the control of FoxO proteins, contributing to skeletal muscle protein loss. FoxO family members regulate atrophy-related ubiquitin ligases *atrogin1/MAFbx, MuRF1, TRIM63, MUSA1, SMART, UBC, USP14*, and *Ube4b*, as well as other genes encoding proteasome subunits, which together are involved in muscle atrophy (Bodine et al., 2001; Gomes et al., 2001; Sandri et al., 2004; Milan et al., 2015). Genes functioning in other pathways connected to authophagy under stress conditions such as unfolded protein response were negatively affected in the FoxO 1,3,4–/– mice, too (Milan et al., 2015). These results strongly support the master regulating role of FoxO transcription factors in muscle atrophy.

#### Nuclear Factor-κB (NF-κB)

Nuclear factor-κB (NF-κB) is an important molecule for multiple cellular responses. Hundreds of genes have been identified that are transcriptionally controlled by NF-κB (Zhang et al., 2017). Recent studies have associated NF-κB activation with the loss of skeletal muscle mass in different physiological and pathological conditions. It has been found that skeletal muscle-specific deletion of IKKβ, an upstream activator of NF-κB signaling, inhibited the expression of MuRF1 E3 ubiquitin ligase. Overexpression of the dominant negative mutant form of IκBα, an inhibitory protein of NF-κB also inhibited the degradation of proteins in muscle. These results suggest the important role of NF-κB activation in muscle-wasting (Bodine et al., 2001; Cai et al., 2004; Cao et al., 2005; Mourkioti et al., 2006).

NF-κB appears to have both activating and inhibitory effects on myogenesis or skeletal muscle formation. It can inhibit myogenic differentiation through transcriptional regulation of cyclin D1 and the transcription factor YinYang1 (YY1) which silences expression of myofibrillar genes (Guttridge et al., 1999; Mitin et al., 2001; Wang et al., 2007). Although NF-κB has been considered as a negative regulator of myogenesis, results from some reports support the role of NF-κB in pro-myogenesis. It has been found that insulin growth factor-II (IGF-II) stimulates NF-κB activation, which further induces the myogenic signaling pathway (Kaliman et al., 1999). These anti- or pro-myogenesis effects on muscles may be determined by a switch between the canonical and non-canonical NF-κB signaling pathway (Bakkar et al., 2008; Bakkar and Guttridge, 2010).

Many studies have described the relationship between NF-κB and autophagy. NF-κB signaling has been shown to be involved in autophagy in a context-dependent manner (Ben-Neriah and Karin, 2011; Hayden and Ghosh, 2012; Salminen et al., 2012; Verzella et al., 2016; Begalli et al., 2017; Bennett et al., 2018). One study has demonstrated that the proinflammatory cytokine TWEAK promotes skeletal muscle atrophy by activating the expression of several autophagy genes including *BECLIN1, LC3B*, and *Atg5* via TRAF6-Mediated NF-κB activation (Dogra et al., 2007). Indeed, NF-κB activation is sufficient to induce the expression of genes involved in or related to the autophagy pathway such as BECLIN1 and the BAG3-HspB8 complex (Copetti et al., 2009; Nivon et al., 2012; Salminen et al., 2012; Rapino et al., 2015). Under starvation conditions, IKKα and IKKβ also stimulate the expression of Atg5, BECLIN1, and LC3 in a NF-κB-independent manner (Comb et al., 2011). On the other hand, NF-κB signaling also has inhibitory effects on autophagy under certain circumstances, which may be mediated by indirect mechanisms. NF-κB signaling may activate mTOR kinase, promote the expression of autophagy inhibitors such as Bfl-1/A1 (a Bcl-2 family member and a BECLIN1 binding partner) and block autophagy inducers such as JNK1, BNIP3, p53, and ROS (Edelstein et al., 2003; Djavaheri-Mergny et al., 2006; Papa et al., 2006; Balaburski et al., 2010; Kathania et al., 2011; Morgan and Liu, 2011; Sarkar et al., 2011; Salminen et al., 2012). These results indicate that the activation of NF-κB signaling in acute stress may induce autophagy while a delayed activation inhibits autophagy. This may represent a protective mechanism from cell death induced by autophagy (Salminen et al., 2012).

These studies demonstrate that NF-κB is a critical regulator of autophagy in skeletal muscle. Although the association of NF-κB with autophagy has been intensively investigated, the majority of these studies focused on cancer. Understanding of the role of NF-κB in muscle autophagy is still limited and future in-depth investigations are needed.

#### Glucocorticoid Receptor (GR) Signaling

Glucocorticoid receptor (GR) signaling has been shown to be associated with protein synthesis and proteolysis in skeletal muscle. GR signaling is crucial for protein breakdown in muscle cells (McGrath and Goldspink, 1982; England and Price, 1995; Hasselgren, 1999; Long et al., 2008; Stahn and Buttgereit, 2008; Sun et al., 2008). During fasting, the expression of poly-ubiquitin mRNA is increased by administration of dexamethasone and the proteolysis pathway is activated in skeletal muscle. Muscle protein breakdown is decreased in adrenalectomized rats, which can be counteracted by glucocorticoid administration (Wing and Goldberg, 1993; Tiao et al., 1996; Mitch et al., 1999). These results suggest that GR signaling is required for protein breakdown in skeletal muscle. Glucocorticoids have been found to stimulate Foxo1 and 3 mRNA in muscle atrophy (Furuyama et al., 2003; Lecker et al., 2004) and activate the expression of UPS related proteins such as atrogin-1, Murf1, and Fbxo30 (Jagoe et al., 2002; Sandri et al., 2004; McLoughlin et al., 2009; Milan et al., 2015). The expression of the autophagy genes Map1lc3b and Bnip3 were induced in both control and glucocorticoid receptor knock-out (GRKO) mice under hypoxia and reduced food intake, but GRKO mice shows a blunted response with impaired expression of Foxo1. These data suggest that glucocorticoid receptor signaling may contribute to autophagy in the context of muscle atrophy through control of gene expression (de Theije et al., 2018). Furthermore, mTOR repressors REDD1 and KLF15 have been identified as direct target genes of the glucocorticoids. Expression of Redd1 induces autophagy and inhibits protein synthesis (Wang et al., 2006; Molitoris et al., 2011; Britto et al., 2014). KLF15 also activates the expression of atrogin-1 and MuRF1 genes and therefore induces skeletal muscle atrophy (Shimizu et al., 2011). One recent study has identified a new mechanism by which a selective NR3C1/glucocorticoid receptor modulator, Compound A (CpdA), has an off-target effect on autophagy. In the classic NR3C1/GR pathway, NR3C1 is recruited to the promoter of *SQSTM1* and other NFE2L2-controlled genes following binding of the GR ligand dexamethasone. In contrast, the transcription factor NFE2L2 is recruited to the promoter of *SQSTM1* by CpdA, suggesting a distinct activating mechanism of autophagy (Mylka et al., 2018). It should be noted that this mechanism has been identified only in mouse bone marrow-derived macrophages. Whether or not the mechanism can be extend to the skeletal muscles needed to be examined.

### Transforming Growth Factor (TGF)-β Signaling

Transforming growth factor (TGF)-β signaling is one of the most important pathways in a variety of physiologic processes. Growth/differentiation factor-8 (GDF-8, myostatin), one of TGF-β family members, has been identified as a skeletal muscle-specific protein from early embryonic development till adulthood. Deletion of the *GDF-8* gene results in a 3 fold increase in skeletal muscle mass, caused by hyperplasia or hypertrophy (McPherron et al., 1997). Multiple mutations in the *GDF-8* gene have been identified in human and mammals, which lead to increased muscle mass (Grobet et al., 1997, 1998; Kambadur et al., 1997; McPherron and Lee, 1997; Szabo et al., 1998; Williams, 2004; Clop et al., 2006; Mosher et al., 2007). The binding of GDF8 to its receptors leads to the phosphorylation of the transcription factor SMAD2/3 and the recruitment of SMAD4 (Sartori et al., 2009). Activation of SMAD2/3 counteracts the inhibitory effect of JunB on FoxO3, contributing to atrophy (Sartori et al., 2009; Welle, 2009; Raffaello et al., 2010). SMAD3 expression also increases the promoter activity of the atrogin-1, MuRF1, and PGC1α, activates the PTEN 3′-UTR and FoxO response element reporters and inhibits the miR-29 promoter activity and mTOR in skeletal muscle leading to protein breakdown and skeletal muscle fiber atrophy (Goodman et al., 2013). These results suggest that GDF8 plays a critical role in muscle atrophy.

TGF-β1 treatment induces phosphorylation of SMAD2 and SMAD3,p62 degradation,LC3β II lipidation and the synthesis of collagen type Iα2 and fibronectin in primary human atrial myofibroblasts (Ghavami et al., 2015). On the other hand, autophagy may be negatively correlated with TGF-β signaling. One recent study has found that the prostaglandin-degrading enzyme, 15-PGDH, is elevated in muscles during aging, which may be responsible for age-related muscle atrophy. Inhibition of 15-PGDH by a small molecule or genetic depletion has beneficial effects on muscle rejuvenation, mediated by a series of events such as increased PGE2, restoration of mitochondrial function, decreased atrogene expression and TGF-β signaling, and increased autophagy (Palla et al., 2021). These results demonstrate the context-dependence of TGF-β-mediated effects in skeletal muscles.

### Other Regulatory Molecules

Other regulatory molecules include mitochondrial proteins OPA1 (optic atrophy 1), DRP1 (Dynamin related protein 1), FGF21 (the fibroblast growth factor 21), and CHMP2B (the charged multivesicular body protein 2B). The mitochondrial dysfunction has been considered as one of the major characteristics of aging process and is associated with muscle loss. OPA1 has been well known for its roles in autophagy and crosstalk with Atg7 (Tezze et al., 2017; Larsson et al., 2019; Zaninello et al., 2020). Muscle-specific Drp1 deletion results in detrimental changes including mitochondrial dysfunction, autophagy impairment, muscle wasting and weakness, suggesting the critical role of mitochondrial dynamics in muscles (Favaro et al., 2019). FGF21 increases glucose uptake and fat utilization in adipocytes, resulting in enhanced mitochondrial oxidation and activation of AMPK. The skeletal muscle-specific deletion of Atg7 stimulates FGF21 expression in an ATG4-dependent manner (Kim et al., 2013). The mice lacking FGF21 showed a decrease in autophagy/lipophagy, which leads to accumulation of lipid and cardiac muscle cell disorganization (Ruperez et al., 2018). Moreover, the general autophagy flux is slightly reduced while the mitophagic flux is significantly decreased in FGF21 knockout mice under starved conditions, and Bnip3 is required for FGF21 induced muscle atrophy and mitophagy (Oost et al., 2019). Other studies showed that increased level of serum FGF21 is associated with multiple metabolic diseases such as muscle atrophy (Staiger et al., 2017; Tezze et al., 2017). Further studies are needed to clarify the physiological and pathological roles of FGF21 in muscle cells (Oost et al., 2019). CHMP2B is a subunit of endosomal sorting complex required for transport-III (ESCRT-III). CHMP2B has been shown to be involved in protein degradation pathways including autophagy and the endosome–lysosome pathway. siRNA-mediated depletion of ESCRT subunits or expression of the CHMP2B C-terminal truncated mutants impairs autophagic degradation, leading to the accumulation of autophagosomes (Filimonenko et al., 2007; Lee et al., 2007).

### Interaction With Ubiquitin/Proteasome Pathway

Skeletal muscle is often subjected to mechanical, heat, and oxidative stress, resulting in cell damage. Proteolysis is required for repair and regeneration in skeletal muscle. Both the ubiquitin/proteasome pathway and autophagy-mediated proteolysis contribute to protein turnover in skeletal muscles. Studies over the past two decades have revealed the connection between these two pathways in skeletal muscles. The transcription factor FoxO3 has been shown to stimulate the expression of many autophagy-related genes and the ubiquitin E3 ligases, atrogin-1/MAFbx (Sandri et al., 2004), as well as lysosomal proteolysis in muscle (Mammucari et al., 2007; Zhao et al., 2007; Bell et al., 2016). Autophagy inhibition results in muscle atrophy, loss-of-force production, myopathy phenotypes, and loss of muscle mass, which is similar to the phenotypes caused by disrupting the functions of atrogin-1 and MuRF1, two atrophy-related ubiquitin ligases, as well as deficiencies in genes involved in different catabolic pathways (Bodine et al., 2001; Baehr et al., 2011). Importantly, both Atrogin-1 and MuRF1 are stimulated in Atg7–/– muscles. Atg7 deletion also results in apoptosis in muscle cells. Muscle-specific Atg5–/– mice showed the same phenotypes as Atg7–/– mice (Raben et al., 2008). Thus the autophagy-lysosome pathway and ubiquitin-proteasome pathway function in parallel downstream of FoxO regulation and may be complementary to each other.

Thus AMPK, mTORC1, mTORC2 and other signaling activities interact with autophagy in the processes of controlling muscle growth, development, size control, atrophy, hypertrophy, and regeneration. The roles of these signalings in the transcriptional regulation of the autophagy pathway have been under intensive investigation in skeletal muscles under normal physiological and diseased pathological conditions. However, the complex and inextricably interwoven network of these signalings and how they are maintained at a finely balanced state remain fully elucidated. Furthermore, how perturbations to genes in these pathways lead to muscle disorders need to be further investigated by human genetics studies and functional studies using model organisms.

## Physiological Roles of Autophagy in Muscle Cells

Accumulating evidence suggests the essential role of autophagy in maintaining cellular homeostasis. Autophagy provides energy and building blocks for metabolism and cellular renewal, controlling the level of amino acids, lipids, carbohydrates and nucleic acids during nutrient deprivation (Tsukada and Ohsumi, 1993; Otto et al., 2003; Scott et al., 2004; Kang et al., 2007). Also, autophagy plays an essential role in intracellular quality control, contributing to the degradation of damaged or aggregated proteins and basal protein turnover. Cells constitutively exhibit a basal autophagy level even under normal growth conditions and autophagy was further induced in response to metabolic stress (Mizushima, 2005). Studies have shown that the inhibition of autophagy results in accumulation of ubiquitinated protein aggregates and inclusion bodies in multiple cell types including muscle cells (Hara et al., 2006; Komatsu et al., 2006; Nakai et al., 2007; Ebato et al., 2008; Jung et al., 2008; Raben et al., 2008; Masiero et al., 2009), as well as abnormalities in mitochondria, peroxisomes, ER and Golgi (Komatsu et al., 2005; Jung et al., 2008). It has now become evident that autophagy involves a highly selective process for the removal of unwanted cellular components and damaged organelles in non-starved cells in addition to its non-selective bulk degradation mechanism (Reggiori et al., 2005; Kraft et al., 2009; Guimaraes et al., 2015).

Many studies have demonstrated the role of autophagy in muscle growth, atrophy, hypertrophy, regeneration and during exercise. In the *Atg7* gene, the cysteine residue encoded by nucleotides in exon 14 is required for activation of substrates. Targeted deletion of exon 14 of Atg7 specifically in adult mice liver impaired autophagosome formation and inhibited the bulk degradation of proteins under fasting condition (Komatsu et al., 2005). In addition, muscle-specific Atg7 knockout mice showed abnormal mitochondria, disorganized sarcomere, reticulum distension, and aberrant concentric membranous structures (Komatsu et al., 2005; Masiero et al., 2009). They exhibited muscle phenotypes such as morphological properties of myopathy, muscle loss and degeneration under catabolic conditions (Masiero et al., 2009). ATG16L1 hypomorphic mice display decreased levels of autophagy, causing a significant reduction in the growth and generation of muscle fibers (Paolini et al., 2018). These data suggest a beneficial role of a basal level of autophagy in maintaining muscle mass and myofiber integrity under physiological conditions (Masiero et al., 2009). Stimulation of autophagy by caloric restriction and exercise may have beneficial effects on lifespan. Impairment of autophagy leads to muscle degeneration and wasting (Masiero et al., 2009; Carnio et al., 2014).

Physical activities have been shown to have an impact on muscle function, which is mediated by autophagy. A proper autophagic flux is required for the degradation of damaged cell organelles and proteins, which provides building blocks and energy during starvation and stress. Exercise and starvation may have the beneficial metabolic effects on human health through BCL2 mediated autophagy (He et al., 2012). On the other hand, excessive autophagy results in atrophy (Grumati et al., 2010). Therefore, an appropriate activation level of autophagy during exercise is critical for muscle homeostasis. Excessive autophagy associated with mutations in the LAMA2 gene resulted in massive muscle wasting. Laminin α2 chain deficiency in mice leads to the increased expression of expression of autophagy-related genes in muscle and the phenotypes of muscle fibrosis, atrophy, and apoptosis, suggesting the pathological role of excessive autophagy (Carmignac et al., 2011; Vainshtein et al., 2014). These findings provide the first evidence that autophagy flux is essential for normal physical activity and defective or excessive autophagy flux leads to muscular dystrophies. This further suggests that the activators of autophagy could serve as potential targets for the treatment of muscular dystrophies (Grumati et al., 2010, 2011; Grumati and Bonaldo, 2012).

Satellite cells are muscle stem cells, which usually reside in a mitotically quiescent state (in G0 phase) and are transcriptionally inactive (Schultz, 1978). The paired domain transcription factor Pax7 is the specific biomarker for all satellite cells (Seale et al., 2000). Once they receive stimuli such as hormones, nutrition, injury or disease, these cells will subsequently begin proliferating to replenish the stem cell pool or differentiating to generate new muscle fibers (Hansen-Smith et al., 1979; Chanoine et al., 1987; Brack and Rando, 2012; Yin et al., 2013). It has been shown that constitutive autophagy is required for satellite cells to maintain their stem cell fitness. Autophagy may provide an energy source for activation by degradation of substrates. SIRT1, a key nutrient sensor, is required for autophagy during satellite cell activation. Either a small molecular inhibitor of SIRT1 or genetic deletion could block autophagy in satellite cells (Tang and Rando, 2014). Failure of autophagy in aged satellite cells or genetically defective cells (such as those from Atg7 knockout mice) results in senescence, oxidative stress and mitochondrial dysfunction, and accumulation of organelles and proteins (Chakkalakal et al., 2012; Cosgrove et al., 2014; Sousa-Victor et al., 2014; Garcia-Prat et al., 2016). Reactivation of autophagy restores their stemness (Garcia-Prat et al., 2016). It has been shown that autophagy is activated in denervation atrophy (Schiaffino and Hanzlikova, 1972) and denervation leads to both a reduction in satellite cell numbers and progressive skeletal muscle atrophy (Schultz, 1978; Rodrigues Ade and Schmalbruch, 1995; Viguie et al., 1997). Thus autophagy is implicated in muscle regeneration. It determines the transition between the quiescence and senescence fate of muscle stem cells. On the other hand, it seems that satellite cells are not the reason for muscle fiber hypertrophy, although they are required for the *de novo* formation of new fibers and fiber regeneration. It is widely accepted that that muscle mass is increased primarily by hyperplasia but not the number of satellite cells (McCarthy et al., 2011; Fukada, 2018). Muscle fiber hypertrophy is functionally normal but regenerative process is significantly reduced in muscle depleted of satellite cells, suggesting that the presence of satellite cells does not determine muscle mass (Amthor et al., 2009; McCarthy et al., 2011; Sambasivan et al., 2011; Jackson et al., 2012; Bachman et al., 2018). In addition, activation of AKT results in an increase in hypertrophic fibers and enhances myofibrillar force without satellite cell proliferation (Blaauw et al., 2009). These findings demonstrate that atrophy may not be attributed to the decline in satellite cell number. Physical exercise and nutrition may be a better treatment for muscle weakness than a stem cell based therapeutical approach (Schwartz et al., 2016).

Autophagy plays a key role in different physiological and pathological processes in heart. Cardiac-specific deletion of Atg5 in mice results in hypertrophy, cardiac dilatation and contractile dysfunction accompanied by disorganized mitochondria, abnormal sarcomere structure and accumulation of misfolded proteins. The Atg5–/– mice develop cardiac dysfunction under stress conditions, demonstrating autophagy activity is required for cardiomyocyte homeostasis (Nakai et al., 2007). In contrast, overexpression of Atg5 stimulates autophagy and increases lifespan in mice (Klionsky et al., 2016). In addition, cardiac-specific Parkin deletion suppresses mitophagy and leads to a lethal cardiomyopathy in developing hearts (Gong et al., 2015).

Autophagy also contributes to ischemia and reperfusion. As discussed above, autophagy is induced by activation of AMPK signaling and inhibition of mTOR signaling. Autophagy was induced by AMPK activation and was suppressed by a dominant negative mutant of AMPK during ischemia, while autophagy was induced by BECLIN1 but not AMPK signaling during reperfusion, suggesting autophagy may play different roles in ischemia and reperfusion (Matsui et al., 2007). Further studies are needed to clarify the underlying mechanism of autophagy during ischemia and reperfusion injury. The roles of autophagy in heart failure vary depending on the context. Heart failure is characterized by cardiac hypertrophy, and can be induced by pressure overload through cardiac autophagy. Heterozygous deletion of BECLIN1, which leads to a reduction of cardiomyocyte autophagy, alleviates cardiac hypertrophy and dysfunction. In contrast, overexpression of BECLIN1 in cardiomyocytes worsens pathologic phenotypes (Zhu et al., 2007). However, phosphorylation of BECLIN1 induced by Mst1 impairs autophagy and induces cardiac dysfunction in heart failure. Mst1 gene deletion activates autophagy and diminishes cardiac remodeling and dysfunction in heart failure (Maejima et al., 2013). These results suggest that the beneficial or detrimental effect of autophagy on heart failure are determined by the specific conditions.

Loss of muscle mass has been found in a variety of diseases such as diabetes, AIDS, sepsis, cardiac disease, and chronic obstructive pulmonary disease (Jagoe and Engelen, 2003; Lecker et al., 2006). These diseases share the same cellular features of excessive protein breakdown which disrupts the balance between anabolism and catabolism, thereby leading to myofiber atrophy (Sandri, 2008). Microarray analyses also demonstrated the up-regulation of autophagy-related genes *LC3* and *GABARAPL1* in muscle wasting induced by different experimental conditions (Lecker et al., 2004). All the studies discussed above demonstrated that the autophagy level is required to be finely tuned and properly regulated to maintain the homeostasis of skeletal muscles during physiological and stressed conditions. Disruption of balanced autophagy leads to the pathogenesis of a variety of muscle disorders, which we discuss in more detail below.

## Pathological Role of Autophagy in Muscle Disorders

Autophagic vacuoles have constantly been observed in skeletal myofibers in diseases like autophagic vacuolar myopathies (AVM) (Malicdan et al., 2008). Muscle disorders cause muscle weakness, muscle wasting and even paralysis, which severely affects patients’ mobility. Environmental factors such as bacterial infection, injury and other diseases like cancers can lead to muscle diseases. Genetic components can also contribute to the development of skeletal muscle disorders (Prior et al., 2007), including mutations in autophagy genes, which are summarized in Table 1.

**TABLE 1:** The mutations in autophagy genes or deregulation of autophagy resulted in skeletal muscle disorders, and the mouse models with impaired autophagy pathway showed the phenotypes of abnormal skeletal muscles.

Gene/locus	Mutation	Inheritance	Diseases	Autophagy association	Clinical features	KO mouse model
DMD	Deletions; Duplications; Point Mutations (PMID: 19937601)	X-linked recessive	Duchenne muscular dystrophy (DMD)	Lower levels of LC3 II and significant accumulation of p62 (PMID: 23152054)	Progressive degeneration of skeletal muscle, impaired heart and respiratory musculature	Resemble human phenotypes (PMID: 6583703; 29479480)
LAMP2	Deletions; Point Mutations (PMID: 20173215; 22695892)	X-linked dominant	Danon disease	Accumulation of autophagic vacuoles (PMID: 10972293)	Heart failure, mental retardation, hypertrophic cardiomyopathy, and proximal muscle weakness	Share many similarities with human phenotypes (PMID: 10972293)
VMA21	Point Mutations (PMID: 31826868)	X-linked recessive	XMEA	excessive autophagy (PMID: 23315026; 27916343)	Slowly progressive muscle weakness	Autophagic myopathy (PMID: 31826868)
GAA	Point Mutations (PMID: 16917947; 14695532)	Autosomal recessive	Pompe disease	Accumulation of glycogen (PMID: 14695532)	hypotonic with large hearts; muscle weakness	Identical with human (PMID: 9384603)
DYSF	Point mutations; Deletions; Insertions (PMID: 18853459: 27602406)	Autosomal-dominant/recessive	LGMD2B	Lipid accumulation (PMID: 24685690)	Wasting; myopathic changes	Mimic human dysferlinopathies (PMID: 23473732)
TRIM32	Point Mutations (PMID: 17994549)	Autosomal recessive	LGMD2H	Bind the autophagy proteins AMBRA1 and ULK1 and stimulate ULK1 activity (PMID: 31234693)	Slowly progressive proximal muscular dystrophy	Resemble human phenotypes (PMID: 19155210)
ATG5				Involved in the extension of the phagophoric membrane in autophagic vesicles (PMID: 17331981)		Small size, small muscle fibers vacuolation and occasional centrally nucleated muscle fibers (PMID: 27693508)
ATG8				Required for fusion of peroxisomal and vacuolar membranes (PMID: 21867568)		Accumulation of ubiquitinated (Ub) proteins and P62/SQSTM1 (PMID: 17580304)

### Duchenne Muscular Dystrophy (DMD)

Duchenne Muscular Dystrophy (DMD) is the most common childhood form of muscular dystrophy. The prevalence is estimated to be 1 in every 3,500–5000 live male births (Emery, 1991; Romitti et al., 2015; Crisafulli et al., 2020). Because DMD is inherited in an X-linked pattern, it primarily affects males, with rare cases in females. Most children with DMD will need a wheelchair by their early teens. Later, heart problems develop into dilated cardiomyopathy with shortness of breath, an irregular heartbeat (arrhythmia), extreme tiredness (fatigue), and swelling of the legs and feet. These heart problems progressively get worse over time, and eventually become life-threatening in most patients (Ryder et al., 2017). DMD had been recognized as a metabolic dysfunction (Dreyfus et al., 1954; Hess, 1965; Di Mauro et al., 1967; Chi et al., 1987; Chinet et al., 1994), and in 1987 the mutations responsible for the disease were first identified in the dystrophin gene on the short arm of the X-chromosome (Koenig et al., 1987). The dystrophin gene is the largest known human gene containing 79 exons and spanning more than 2,200 kb, accounting for almost 0.1% of the entire human genome (Gao and McNally, 2015). The most common type of mutations in this gene are deletions of one or more exons, accounting for 60–70% of cases. The other mutations include small deletions, insertions, exonic duplications, splicing mutations, and point mutations (Flanigan et al., 2009; Tuffery-Giraud et al., 2009). These mutations may result in reading frame shift and subsequently produce truncated proteins with premature stop codons.

It has been shown that autophagy was impaired in DMD patients and mdx mouse models which closely mimic the human disease (De Palma et al., 2012). In mdx mice, mTOR was constitutively activated, leading to the down-regulation of LC3, Atg12, Bnip3 and Gabarapl1 at the molecular level. At the same time, a long-term low-protein diet treatment reactivated autophagy through inactivation of AKT (De Palma et al., 2012). Consistent with these observations in mdx mice, the expression and phosphorylation levels of AKT in the skeletal muscles and cardiac muscles of DMD patients were largely increased (Pichavant et al., 2011). In addition to the AKT signaling pathway, ROS generation by CYBB/NOX2 also led to autophagy deficiency in skeletal muscles of the mdx mice. Simvastatin treatment suppressed the generation of ROS and increased autophagy signaling. These findings suggest that autophagy may serve as a novel therapeutic target for DMD patients (De Palma et al., 2012; Whitehead et al., 2016).

It has also been found that P2RX7 (the purinergic receptor P2X, ligand-gated ion channel, 7) is stimulated in mdx mouse myoblasts and myofibers (Young et al., 2012). The large-pore formation of P2RX7 and HSP90 are required for the ATP-evoked autophagic death of dystrophic muscles (Young et al., 2015). Administration of Coomassie Brilliant Blue, the P2RX7 antagonist, leads to a reduction of degeneration-regeneration cycles in mdx mice, suggesting P2RX7 may act as a potential drug target for the treatment of the disease (Young et al., 2012; Bibee et al., 2014; Sinadinos et al., 2015).

Another molecule implicated in the pathogenesis of mdx mice is TNF receptor-associated factor 6 (TRAF6), which has a role in maintaining skeletal muscle mass. TRAF6 is upregulated in skeletal muscle of mdx mice. Inhibition of autophagy through the targeted deletion of TRAF6 in mdx mice appears to preserve skeletal muscle mass at the initial stage but exaggerates dystrophic phenotype at the late stage, suggesting the opposing effect of autophagy on skeletal muscles in mdx mice (Hindi et al., 2014).

Other approaches increasing autophagy or protein quality control have also been considered as potential therapeutic approaches for DMD. Peroxisome proliferator-activated receptor gamma coactivator 1-alpha (Pgc-1α) gene transfer results in an increase in Lc3 and Atg12 in mdx mouse muscles, indicating a beneficial effect of autophagy on dystrophic skeletal muscle (Hollinger et al., 2013). One recent study has demonstrated that the modulations of protein quality control mechanisms have been established in undifferentiated myoblasts derived from DMD patients, but misfolded/aggregated proteins are determined to take the path to autophagy rather than to proteasome. This change is caused by a switch from BAG1 to BAG3, NFκB activation, and up-regulation of BAG3/HSPB8 complexes. Restoration of the established mechanism of protein quality control may be a potential therapeutical target for DMD treatment (Wattin et al., 2018).

### Ullrich Muscular Dystrophies and Bethlem Myopathy

Collagen VI is a major extracellular matrix protein of skeletal muscle involved in cell adhesion and membrane stabilization. Collagen VI has been shown to be associated with of numerous physiological and pathological conditions. A number of mutations in Collagen VI have been identified in disorders of muscular dystrophy like Ullrich muscular dystrophies and Bethlem myopathies (Bonnemann, 2011). It has been suggested that reduced autophagocytic flux played a critical role in the pathogenesis of collagen VI deficiency (Grumati et al., 2010). Autophagy induction is impaired after physical exercises in collagen VI null mice, which has a detrimental effect on muscles (Grumati et al., 2011). Several molecular changes have been found in collagen VI null mice, such as reduced autophagosomes and LC3 lipidation, and impaired induction of BECLIN1 and Bnip3 (Grumati et al., 2010).

### Autophagic Vacuolar Myopathies (AVM)

AVM is a group of rare genetic disorders that share common histopathological features on muscle biopsy with an excess of autophagic vacuoles and sarcolemmal characteristics (Munteanu et al., 2015). Mutations in genes related to autophagy have also been identified among patients with the spectrum of AVM (Table 1).

#### Danon Disease

Danon disease, originally named as lysosomal glycogen storage disease with normal acid maltase, is the best-known AVM and is characterized by weakening of cardiomyopathy, weakening of myopathy, and neurological phenotypes like intellectual disability. Danon disease is caused by mutations in the gene encoding the Lysosome-associated membrane protein 2 (LAMP2), a membrane glycoprotein known to be related to autophagy. LAMP2 may play a role in controlling cell–cell or cell-extracellular matrix adhesion and maturation of autophagic vacuoles in addition to maintaining lysosomal structural integrity (Lippincott-Schwartz and Fambrough, 1986; Carlsson et al., 1988; Saitoh et al., 1992; Lichter-Konecki et al., 1999; Eskelinen, 2006). There are different splicing forms of LAMP2 in various tissues (Konecki et al., 1995), among which LAMP2A is the only isoform with positive amino acid residues at the carboxyl terminus tail responsible for substrate binding (Cuervo and Dice, 2000). A recent study has demonstrated that the Asn175 site at the linker region between N- and C-terminal subdomains of LAMP2 is critical for its role in autophagy. Loss of glycosylation at the Asn175 disrupts the interaction between Lamp2 and galectin-9 protein, which impairs endolysosome/lysosome function and cargo degradation (Sudhakar et al., 2020).

A variety of mutations in LAMP-2 have been reported in patients with Danon disease. Most of these mutations are nonsense or frameshift mutations, which result in truncated protein products (Brambatti et al., 2019; Gurka et al., 2020). Lack of the transmembrane and cytoplasmic domains at the C-terminal tail abolishes its function as a lysosomal membrane protein (D’Souza et al., 2014). Ample evidence indicates that deficiency of LAMP-2 causes mistargeting of certain lysosomal enzymes and impaired capacity for lysosomal degradation (Tanaka et al., 2000; Eskelinen et al., 2002), leading to disrupted phagosomal maturation, autophagosome-lysosome fusion, and accumulation of autophagosomes and resulting in myopathy and cardiomyopathy (Tanaka et al., 2000; Saftig et al., 2008). In contrast, overexpression of Lamp2 can alleviate autophagic flux blockade likely due to stimulation of cathepsin trafficking, which may improve cardiomyocyte resistance to lysosomal cell death (Cui et al., 2020).

#### X-Linked Myopathy With Excessive Autophagy (XMEA)

X-linked myopathy with excessive autophagy (XMEA) is a rare disorder characterized by childhood onset of weakness and wasting, mainly in the proximal muscles of the lower extremities. Although the muscles including the anterior thigh, the ankle dorsiflexors, the hip girdles and the shoulder are affected, other organs, such as the heart appear to be normal in the majority of patients (Kalimo et al., 1988). Serum creatine kinase levels are dramatically elevated in the patients. The morphological abnormalities in muscle cells are easily observed with an optical microscope (Kalimo et al., 1988; Villanova et al., 1995; Minassian et al., 2002; Crockett et al., 2014).

XMEA is caused by mutations in the VMA21 gene at Xq28 encoding a chaperone protein for the lysosomal vacuolar ATPase (Ramachandran et al., 2013). Vacuolar ATPases are rotary proton pumps across the plasma membrane regulating the pH of intracellular organelles. VMA21 is required for the proper assembly of multiple proton pump subunits (Forgac, 2007). Loss of VMA21 disrupted the interaction between the major proteolipid subunit of V0 and another V0 subunit, Vph1p during assembly (Malkus et al., 2004). Loss of appropriate VMA21 activity results in the formation of autophagic vacuoles with sarcolemmal features (Crockett et al., 2014). Mechanistic study showed that reduced VMA21 level leads to increased lysosomal pH and decreased lysosomal degradative ability. Meanwhile, feedback upregulation of autophagosome formation and inhibition of the mTORC1 pathway results in accumulation of ineffective autolysosomes, cell vacuolation and tissue atrophy (Ramachandran et al., 2013). A recent study of *in vitro* cultured patient-derived myoblast cells revealed the possible mechanism by which VMA21 mutation triggers autophagy abnormity may contribute to XMEA development. This study demonstrated VMA21 mutation-associated autophagy defect leads to uncontrolled myoblast fusion and altered myoblast differentiation, which produced functionally inferior muscle cells. This observation also explains why muscles are the predominantly involved tissue in XMEA disorder (Fernandes et al., 2020).

Skeletal muscle of XMEA patients with VMA21 mutations is the main affected tissue, which may be due to the difference in V-ATPase demand between skeleton muscles and other organs (Ramachandran et al., 2013; Munteanu et al., 2015; Saraste et al., 2015). Additional study has identified a mutation (c.164-6T > G) resulting in much lower VMA21 expression and V-ATPase activities than those in classical XMEA; this patient does have comparatively mild cardiac hypertrophy (Munteanu et al., 2017). Another study revealed that the mutations c.10C > T, p.Arg18Gly and p.Asp63Gly in VMA21 were implicated in a congenital disorder of glycosylation with autophagic liver disease (Cannata Serio et al., 2020).

#### Pompe Disease

Pompe disease, also called type II glycogen storage disease, is a rare, autosomal recessive metabolic disorder characterized by α-glucosidase deficiency leading to accumulation of glycogen in the lysosomes (Dasouki et al., 2014). This disease has been classified into two subtypes according to the disease onset age. Infantile-onset Pompe disease is the severe subtype with which children develop symptoms under 12 months of age. This subtype is characterized by heart muscle malfunction or severe breathing problems, typically leading to death due to cardiac failure or respiratory abnormality within the first year of life without treatments. Late-onset Pompe disease is usually a mild form in which symptoms may begin at any time from late childhood to adulthood and progress more slowly than those in infantile-onset Pompe disease (Dasouki et al., 2014; Kohler et al., 2018).

Pompe disease is caused by the mutations in GAA gene encoding acid alpha-glucosidase which breaks down glycogen to glucose in the lysosome. The GAA gene is located on the long arm of chromosome 17 (17q25.2-q25.3) and contains 20 exons spanning 18.3 kb. Over 600 mutations in the GAA gene have been identified in patients with Pompe disease and missense mutations are the most common type (Pompe variant database^1^). Although most of the GAA mutations are rare, the variant rs386834236 (c.-32-13T > G) is common among Caucasian patients. The variant leads to the spicing out of the exon (Huie et al., 1994; Raben et al., 1996). The c.525delT and the c.2481 + 102_2646 + 31del mutations are overrepresented in the Dutch population, both of which lead to a reading frameshift with an early stop codon (Peruzzo et al., 2019). The severity of the disease depends on the degree of enzyme deficiency determined by the nature of the mutations in both alleles.

Dysfunction of acid alpha-glucosidase results in glycogen accumulation in the lysosomes, followed by lysosomal rupture in cardiac and skeletal muscles, leading to severe myofibril loss (Griffin, 1984; Thurberg et al., 2006). Autophagic accumulation in skeletal muscle was observed in Pompe patients (Fukuda et al., 2006) and further immunostaining with LAMP1 and LC3 in muscle fibers revealed extensive accumulation of autophagosomes, clustering of late endosomes and broken lysosomes (Raben et al., 2007; Raben et al., 2009). In a mouse model of Pompe disease, muscle-specific loss of GAA results in an autophagy defect due to the impaired autophagosomal–lysosomal fusion, and subsequently the phenotype of muscle atrophy (Murrow and Debnath, 2013). The enzyme acid alpha-glucosidase is produced as an inactive precursor which has to be glycosylated in the ER and phosphorylated in Golgi before maturation through proteolysis in the endosome/lysosomes (Moreland et al., 2005; Meena and Raben, 2020). Any mistake during this process would result in an acid alpha-glucosidase deficiency. The defective autophagic pathway in patients with Pompe Disease also leads to mitochondrial abnormalities detected in muscle biopsies. A number of therapeutic approaches have been developed to improve enzyme replacement for Pompe disease (Spampanato et al., 2013; Tian et al., 2019).

### Limb Girdle Muscular Dystrophy Type 2B (LGMD2B) and Miyoshi Muscular Dystrophy 1 (MMD1)

LGMD2B is an autosomal recessive disease characterized by proximal muscle weakness and wasting affecting shoulder girdles and pelvises with slow progression (Aoki, 1993). MMD1 is characterized by distal muscle weakness affecting the upper and lower limbs (Miyoshi et al., 1986). Both LGMD2B and MMD1 are caused by mutations in the gene dysferlin (DYSF) (Liu et al., 1998), which contains 55 exons, spanning a 230 kb genomic region located on chromosome 2p13.2. The protein product dysferlin is a type II transmembrane protein expressed mainly in muscle sarcolemma. It is involved in muscle contraction, calcium-mediated membrane fusion, and membrane regeneration. Dysferlin contains seven C2 domains (C2A to C2G) and two DysF domains, among which the C2A domain is responsible for Ca^2+^ and phospholipid binding.

Different types of DYSF mutations have been uncovered in LGMD2B and MMD patients, and the most frequently observed pathogenic variant is rs28937581 (c.2997 G > T; p.Trp999Cys) located in a DysF domain (Izumi et al., 2020). Crystal structures of the DysF domain indicate that mutations like p.Arg959Trp, p.Trp999Cys, and p.Arg1046His may disrupt an aromatic/arginine stack motif, leading to instability of the protein (Sula et al., 2014). DYSF mutations result in mRNA instability and degradation which further stimulates the autophagy and proteasome pathways (Barthelemy et al., 2011). Muscles from dysferlinopathy patients show elevated MuRF-1, LC3-II, p62/SQSTM1 and Bnip3 levels, and fiber atrophy phenotypically. Protein aggregates are found in these muscle fibers which is stained positive for p62. These observations suggest that altered proteasomal degradation of mutant dysferlin and autophagy level (Fanin et al., 2014). Insufficient membrane fusion and accumulation of vesicles have been observed in SJL/J mice carrying a splice-site mutation in the Dysf gene, implicating the role of dysferlin in autophagy process (Hino et al., 2009). Excessive mutant dysferlin may have a detrimental effect on muscle cells. Mutant dysferlin aggregates in the ER and induces autophagosome formation through eukaryotic translation initiation factor 2a (eIF2α) phosphorylation, therefore autophagy/lysosome is an important alternative to the ubiquitin proteasome system for the degradation of excess mutant dysferlin in ER-associated protein degradation (ERAD). Defects in the autophagy pathway lead to a more severe phenotype, exemplified by the increased aggregation of mutant dysferlin in the ER caused by Atg5 deficiency and dephosphorylation of eIF2α (Fujita et al., 2007). In myocytes developed from induced pluripotent stem cells from a patient carrying p.Trp999Cys mutation, nocodazole treatment increases dysferlin levels and improves membrane resealing, suggesting that dysferlin degradation may be a potential drug target for the treatment of dysferlinopathy (Kokubu et al., 2019).

### Limb Girdle Muscular Dystrophies Type 2H (LGMD2H)

LGMD2H is a relatively mild form of myopathies caused by mutations in the gene encoding Tripartite motif-containing protein 32 (TRIM32), a well-known E3 ubiquitin ligase (Shieh et al., 2011). A recent study showed that TRIM32 is required for autophagy induction in response to atrophic stimuli *in vivo* in mouse models. At the molecular level, TRIM32 interacts with the autophagy proteins AMBRA1 and ULK1, and thereby stimulates ULK1 activity through K63-linked ubiquitin chains. The pathogenic TRIM32 mutant p.Val591Met found in LGMD2H patients disrupts the interaction between TRIM32 and ULK1, therefore inhibiting autophagy induction, which leads to the exacerbated atrophy exhibited by an elevated level of ROS production and MuRF1 expression (Di Rienzo et al., 2019a, b). Another study showed that the disease-associated mutants p.Pro130Ser, p.Asp487Asn, p.Arg394His, and p.Val591Met inhibit autophagic degradation of p62/SQSTM1 in muscle cells (Overa et al., 2019). These studies link the stress of muscle inactivity caused by defects in ubiquitination to the impaired induction of autophagy machinery.

### Sporadic Inclusion Body Myositis (sIBM)

Sporadic inclusion body myositis (sIBM) is the most common form of acquired myopathy among adults aged over 50 years. Similar to Alzheimer’s disease, sIBM patients present the pathological feature of sporadic inclusion body myositis, which is characterized by abnormal accumulation of amyloid precursor protein (APP) and its proteolytic fragment, amyloid-β (Aβ). It has been shown that APP colocalizes with Atg8/LC3, and APP/beta-amyloid-containing autophagosomes are increased in muscle fibers of sIBM muscle biopsies, suggesting that the autophagy pathway is essential for the degradation of APP/beta-amyloid (Lunemann et al., 2007). sIBM tissues demonstrate damaged myofibres with obvious accumulation of p62/SQSTM1 and TDP-43 (Weihl et al., 2008; Salajegheh et al., 2009). Mechanistic study shows that the altered binding of the p62-ubiquitinated protein complex to LC3 in sIBM patients results in the early termination of autophagy at initiation stage, resulting in p62 protein aggregates (Nakano et al., 2017). Study of an animal model shows that resistance exercise may induce a hypertrophy signal, and alleviate autophagy and muscle atrophy, suggesting a preventive approach for sIBM (Jeong et al., 2017).

## Conclusion and Future Directions

In summary, skeletal muscle is the most abundant tissue type in the human body, maintaining a finely tuned metabolism balance between catabolic and anabolic processes. Autophagy is an essential catabolic process responsible for the degradation of proteins and cellular organelles through the autophagosome-lysosome system with the involvement of evolutionally conserved ATG proteins. Autophagy plays a central role in maintaining cellular homeostasis through complex interactions with diverse signaling pathways under physiological conditions and metabolic stress in skeleton muscles. A basal level of autophagy is required for homeostasis in skeletal muscles due to the frequent turnover of protein and cytoplasmic components. Both deficient and excessive autophagy result in a pathological cascade and lead to muscular weakness and atrophy symptoms. Abnormal autophagy levels may also contribute to cell damage. Both increased formation of autophagosomes and inhibition of lysosome-autophagosome fusion cause myopathy. Mutations in autophagy genes and deregulation of autophagy pathways have been identified as one of the major causes of various muscle disorders.

Although advances have been made in understanding the role of autophagy in skeletal muscle disorders over the past two decades, much remains to be elucidated regarding the molecular mechanisms underlying abnormal autophagy activity in skeletal muscle disorders. Traditional experimental techniques employed in studies of other cell and tissue types can be utilized further to examine the contribution of autophagy to skeletal muscle disorders, especially to investigate whether the contribution is cell-type and tissue-type specific. Multi-omics techniques achieved astonishing advancement during the past 10 years and have been widely applied to the studies of complex human diseases. Future studies of skeletal muscle diseases can adopt these omics approaches. High-throughput sequencing at the DNA level can be applied to identify additional causal mutations, which may help to elucidate the underlying mutation landscape of skeletal muscle diseases. RNA sequencing of patient biopsies will generate altered expression profiling under different disease conditions compared to normal physiological conditions. For skeletal muscle diseases, proteomics studies to examine protein modification changes such as phosphorylation and ubiquitination levels, and metabolomics studies are particularly important. As we can see from the literature that we have reviewed, skeletal muscle disorders are clearly related to cellular metabolism. The change in autophagy and its interaction with other signaling pathways is reflected not only in gene expression but also in post-translational modification at the protein level. The application of multi-omics techniques will give a broader view of the influence of autophagy on skeletal muscle disorders and a deeper understanding of the contribution of autophagy to the pathogenesis of the diseases. Further understanding of the molecular and biochemical mechanisms underlying the role of autophagy in skeletal muscle disorders will help to develop new interventional and therapeutic strategies for the diseases.

## Author Contributions

YW and JL were responsible for conception and design of study. QX, XH, JH, YZ, and MM conducted literature search, summarization, and drafted the manuscript. YW and JL revised the manuscript. All authors have read and approved the manuscript.

## Conflict of Interest

The authors declare that the research was conducted in the absence of any commercial or financial relationships that could be construed as a potential conflict of interest.

## Footnotes

1 www.pompevariantdatabase.nl
